# The Paul Glaucoma Implant: a systematic review of safety, efficacy, and emerging applications

**DOI:** 10.1007/s00417-025-06861-2

**Published:** 2025-05-28

**Authors:** Matteo Mario Carlà, Gloria Gambini, Francesco Boselli, Lorenzo Hu, Agnese Maria Perugini, Emanuele Crincoli, Fiammetta Catania, Laura De Luca, Stanislao Rizzo

**Affiliations:** 1 Ophthalmology Department, “Fondazione Policlinico Universitario A. Gemelli, IRCCS”, 00168 Rome, Italy; 2 Ophthalmology Department, Catholic University “Sacro Cuore”, 00168 Rome, Italy; 3 Department of Ophthalmology, Hopital Fondation Adolphe De Rothschild, Paris, France; 4 Ophthalmology Clinic, Department of Biomedical Sciences, University of Messina, Messina, Italy

**Keywords:** Paul glaucoma implant, PGI, Glaucoma draining device, Hypotony, Tube erosion

## Abstract

**Purpose:**

The Paul Glaucoma Implant (PGI) is a novel non-valved glaucoma drainage device designed to lower intraocular pressure (IOP) in patients with refractory glaucoma. This systematic review aims to evaluate the current evidence on safety, efficacy, and emerging applications of PGI implantation.

**Methods:**

A systematic literature search was conducted following PRISMA guidelines across PubMed/Medline, Embase, Cochrane, Google Scholar, and Web of Science databases up to April 2025. Quality assessment was performed using the NIH Quality Assessment Tool for Before-After Studies. Data on patient demographics, surgical techniques, IOP outcomes, success rates, and complications were extracted and analyzed.

**Results:**

Eighteen studies (946 eyes) met the inclusion criteria. The PGI consistently demonstrated significant IOP reduction across various studies and glaucoma subtypes. Mean IOP reductions ranged from 14.8 mmHg to 19.1 mmHg, depending on follow-up duration and patient characteristics. Complete success rates ranged from 38.4% to 75%, while qualified success rates were consistently high, reaching up to 93.2%. The PGI's unique design, featuring a smaller inner tube diameter (0.127 mm), is thought to contribute to a lower risk of hypotony compared to other non-valved implants. Three comparative studies with other GDDs reported similar success rates with potentially fewer early complications. Quality assessment revealed moderate-to-good quality evidence, with limitations including short follow-up periods and predominance of uncontrolled studies.

**Conclusions:**

The PGI is emerging as a safe and effective surgical option for treating refractory glaucoma. Its smaller tube diameter and large surface area endplate contribute to efficient IOP control with a potentially lower risk of hypotony.

## Abstract (highlights)

**Key messages:**

*****What is known***:**

- Glaucoma drainage devices (GDDs) are an established treatment option for refractory glaucoma.

*****What is new***:**

- The Paul Glaucoma Implant (PGI) is a novel, non-valved GDD with a smaller lumen that has shown promising results in lowering intraocular pressure (IOP) in several glaucoma etiologies until 3 years of follow-up, showing lower rates of post-operative hypotony due to its intrinsic design.
- PGI has showed the propensity to form double-layered blebs, which are associated with lower IOP, and it tends to create larger blebs compared to other GDDs.
- Recent comparative studies with Ahmed Glaucoma Valve (AGV) and Baerveldt Glaucoma Implant (BGI) have shown comparable efficacy with potentially improved safety profiles, though long-term results are still pending.

## Introduction

Glaucoma, a leading cause of irreversible blindness worldwide, affects millions of individuals, imposing a significant public health burden. The hallmark of this condition is elevated intraocular pressure (IOP), which damages the optic nerve, leading to progressive vision loss [1, 2]. Controlling IOP remains the cornerstone of glaucoma management, aiming to halt or slow disease progression and preserve vision [2]. While medical therapy often constitutes the first-line approach, surgical interventions become necessary when medications fail to achieve target IOP or in cases of advanced disease [1].

In recent years, the gold standard trabeculectomy has been flanked by numerous minimally invasive options [3–5]. However, in the setting of refractory glaucoma, glaucoma drainage devices (GDDs) have emerged as the most valuable treatment tool, offering an alternative pathway for aqueous humor outflow [6]. Commonly employed GDDs include the Ahmed Glaucoma Valve (AGV) and the Baerveldt Glaucoma Implant (BGI), each with its own mechanism of action and potential complications. Surely, these devices are not exempt from complications, mostly characterized by the risk of postoperative hypotony, choroidal detachment and late tube exposure [7].

In recent years, the Paul Glaucoma Implant (PGI), a novel non-valved GDD, has been introduced as a potential solution for managing moderate to advanced glaucoma [8]. The PGI distinguishes itself by its smaller internal tube diameter (0.127 mm), acting as an intrinsic flow restrictor while maintaining a surface area comparable to the BGI (342 mm.^2^). This design aims to mitigate the risk of early postoperative hypotony, a common concern with non-valved GDDs [8, 9].

While several studies have reported outcomes of PGI implantation, a comprehensive synthesis of the current evidence is lacking. Such a synthesis is essential to guide clinical decision-making and identify areas requiring further investigation. Therefore, this systematic review aims to evaluate the safety and efficacy of PGI, synthesizing data from all eligible studies and including recently published comparative trials.

## Methods of literature review

This systematic review was conducted in accordance with the Preferred Reporting Items for Systematic Reviews and Meta-Analyses (PRISMA) guidelines.

### Search strategy

An extensive search of studies was conducted on PubMed/Medline, Embase, Cochrane, Google Scholar and Web of Science databases, including all articles until January 2025. We used various combinations of key terms related to “Paul Glaucoma Implant”, “PGI”, “Glaucoma”, “Baerveldt Glaucoma Implant”, “Ahmed Glaucoma Valve”, “non-valved glaucoma implant”, “non-valved glaucoma device”, “glaucoma draining device”, connected by Boolean operators. Reference lists of eligible articles were reviewed as well.

### Eligibility criteria

Inclusion criteria were: (1) studies involving human subjects who underwent PGI implantation; (2) studies reporting clinical outcomes including IOP reduction, success rates, and/or complications; (3) studies in any language (with translation if needed); (4) randomized controlled trials, non-randomized controlled trials, prospective or retrospective cohort studies, and case series or case reports.

Exclusion criteria were: (1) review articles, letters, commentaries, or editorials; (2) in vitro or animal studies; (3) studies with incomplete or duplicated data; (4) studies not providing specific outcomes related to PGI efficacy or safety.

### Study selection and data extraction

Two independent reviewers (MMC and GG) screened titles and abstracts of all identified articles. Full texts of potentially relevant articles were then evaluated against the eligibility criteria. Disagreements were resolved through discussion with a third reviewer (FB).

Data extraction was performed using a standardized form and included: (1) study characteristics (author, year, design, sample size, follow-up duration); (2) patient demographics and baseline characteristics; (3) surgical technique details; (4) outcome measures (IOP, medication use, success rates using various definitions); (5) complications and adverse events.

### Quality assessment

Quality assessment of included studies was performed using the Quality Assessment Tool for Before-After (Pre-Post) Studies with No Control Group developed by the National Institutes of Health for non-randomized studies. For comparative studies, the Risk of Bias In Non-randomized Studies of Interventions (ROBINS-I) tool was applied. Quality assessment was performed independently by two reviewers (EC and FC), with discrepancies resolved by consensus.

### Data synthesis

Due to the heterogeneity in study designs, populations, and outcome definitions, a narrative synthesis was conducted rather than a meta-analysis. Studies were grouped by design (comparative vs. non-comparative) and patient populations (adult vs. pediatric, primary vs. secondary glaucoma) to facilitate comparison.

**Fig. 1:**
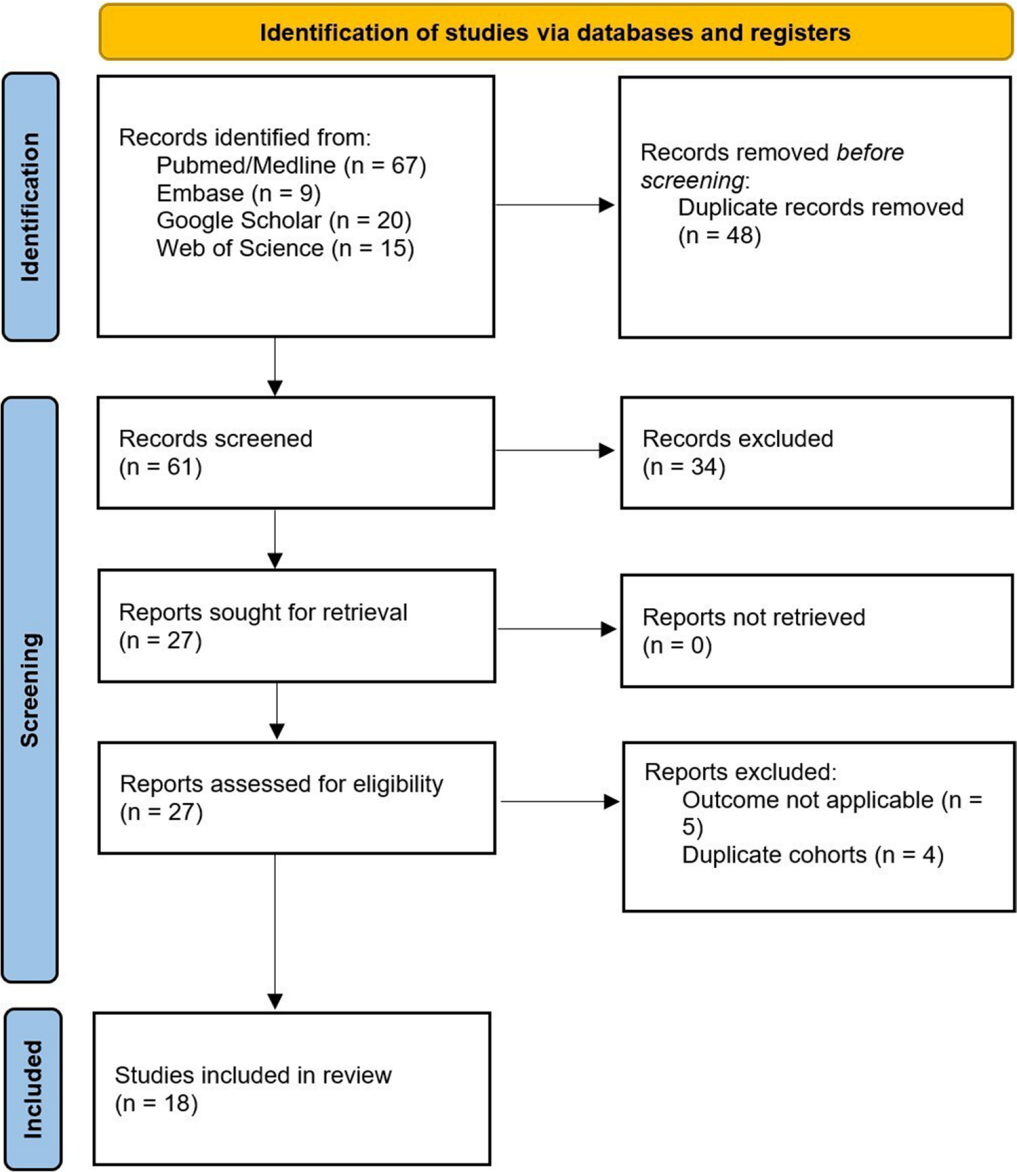
Preferred Items for Systematic Reviews and Meta-Analyses (PRISMA) flow diagram, adapted for the current research

Figure 1 illustrates the PRISMA flow diagram for study selection.

## Surgical indications

PGI implantation is primarily indicated for the management of glaucoma that is refractory to medical treatment and/or previous surgical interventions [8]. This device is considered a suitable option for patients with moderate to advanced glaucoma who require lower target IOP levels to halt disease progression. Its smaller internal lumen diameter makes it particularly appealing for individuals at higher risk of surgical failure and hypotony-related complications [6].

Among published studies, PGI was effectively employed in cases of refractory primary open-angle glaucoma (POAG), pseudoexfoliative glaucoma (PXG), secondary glaucomas, including uveitic glaucoma, neovascular glaucoma and glaucoma following congenital cataract surgery (GFCS) and also in pediatric glaucoma [8–19], since the smaller size of the PGI tube could be theoretically advantageous in pediatric patients, as it may cause less endothelial damage and reduce the risk of corneal decompensation, a major concern in this population.

Additionally, recent publications have explored the use of PGI in cases of secondary glaucoma following vitreoretinal surgery and for managing aphakic glaucoma, broadening the potential applications of this device.

Technical features of PGI and comparison with BGI and AGV is available in Table 1.

**Table 1:** Comparisons between technical data and potential advantages and disadvantages of three glaucoma draining devices: Paul Glaucoma Implant, Baerveldt Glaucoma Implant and Ahmed Glaucoma Valve

Feature	Paul Glaucoma Implant (PGI)	Baerveldt Glaucoma Implant (BGI)	Ahmed Glaucoma Valve (AGV)
Type	Non-valved	Non-valved	Valved
Plate Surface Area	342 mm^2^	350 mm^2^	184 mm^2^
Plate Thickness	0.95 mm	0.9 mm	1.0 mm
Tube Outer Diameter	0.467 mm	0.64 mm	0.64 mm
Tube Inner Diameter	0.127 mm	0.3 mm	0.3 mm
Potential advantages	- Reduced risk of hypotony- Smaller tube size may be less disruptive to the anterior chamber - Larger blebs associated with lower IOP	- Effective long-term IOP lowering- Large plate surface area	- Lower risk of early hypotony due to valve - Immediate flow control without need for ligation techniques
Potential disadvantages	- Newer device with less long-term data available - Potentially higher risk of tube exposure due to larger blebs	- Higher rate of hypotony-related complications - Requires flow restriction techniques in early postoperative period	- Less effective long-term IOP lowering compared to BGI - Smaller plate surface area - Higher risk of encapsulation and hypertensive phase

## Surgical technique

The surgical procedure for PGI implantation involves a standardized protocol, aimed at ensuring consistent outcomes and minimizing variability. A fornix-based conjunctival incision is created, followed by careful dissection of the conjunctiva and Tenon's capsule, exposing the sclera. Meticulous hemostasis is crucial at this stage to ensure clear visualization and prevent postoperative bleeding. In some cases, the surgeon may choose to apply Mitomycin C (MMC**) (0.2–0.4 mg/ml)** to the scleral bed for a short duration, typically 90 s, followed by thorough rinsing with saline. The use of MMC, however, is not standardized for PGI, even if employed in several reports in literature [8, 9, 18, 20].

The PGI endplate, typically positioned in the superotemporal quadrant, is secured under the rectus muscles, with its anterior part sutured at around 8–10 mm from the limbus. After injections of viscoelastic to deepen the anterior chamber, a scleral tunnel is then fashioned starting 2 mm from the limbus and employing a 25- or 26-gauge needle, keeping a direction parallel to the iris. Successively the tube, after being trimmed to the appropriate length, is carefully introduced bevel-up into the anterior chamber, just above the iris plane to avoid contact with the corneal endothelium. To cover the exposed tube, a patch of bovine pericardium Tutopatch or donor corneal graft is then used, and the conjunctiva and Tenon are finally sutured at the limbus.

Additional techniques were described to limit the outflow of PGI, such as partial ligation of the tube with a 7–0 Vicryl suture halfway around the circumference [9], or placement of a ripcord suture around the partially ligated tube, which can be removed later to increase aqueous flow if needed [12, 21]. Finally, Vallabh et al. described the use of an intraluminal stent, often made of Prolene or nylon, to further modulate aqueous flow and prevent early hypotony, being then possibly removed in the late post-operative period when the bleb has formed and stabilized [20].

In cases of neovascular glaucoma, Olgun et al. [22] reported on the adjunctive use of intraoperative MMC (0.4 mg/ml concentration for 2–3 min in the subconjunctival space), which was associated with a lower frequency of additional interventions (10.4% vs 22.7% in the group without MMC), though larger studies are needed to confirm this observation.

## Early and late results

Of the 18 studies included in this systematic review with a total of 946 eyes, 15 were non-comparative cohort studies (prospective or retrospective) and 3 were comparative studies (2 comparing PGI with BGI and 1 comparing PGI with AGV). A summary of studies reporting PGI outcomes is available in Table 2.

**Table 2:** Summary of the effectiveness and post-operative complications of Paul Glaucoma Implant among different studies

Study (First Author, Year)	Number of Eyes	IOP Preoperative (mm Hg)*	IOP Postoperative (mm Hg)*	Follow-up (Months)	Complete Success Rate (≤ 21 mmHg)	Qualified Success Rate (≤ 21 mmHg)	Reported Complications and Prevalence
Koh et al. 2020	74	23.1 (SD 8.2)	13.2 (SD 3.3)	12	68.9%	93.2%	Shallow anterior chamber (14.9%), hypotony requiring intervention (9.5%), tube shunt occlusion (6.8%), tube exposure (4.1%), endophthalmitis (1.4%)
Vallabh et al. 2021	99	28.1 (SD 9.0)	13.3 (SD 4.4)	12 (52 patients)	38.4%	90.1%	Hypotony (2%), hyphaema (4%), cystoid macular edema (2%), recurrent inflammation (2%), corneal graft failure (2%)
José et al. 2022	24	31.4 (SD 10.0)	12.5 (SD 4.3)	12	33%	75%	Tube exposure (4%), cataract (33%), fibrous tissue ingrowth (4%), hyphema (4%), iris synechiae (13%), choroidal detachment (8%), strabismus (13%)
Tan et al. 2022	45	19.8 (SD 6.3)	13.9 (SD 3.7)	24	71.1%^α^	82.2%^α^	Shallow anterior chamber (22.2%), hypotony requiring intervention (8.9%), tube shunt occlusion (8.9%)
Weber et al. 2023**	45	26.1 (range 7–48)	12.0 (range 3–24)	12	73.3%	95.6%	Transient hypotony (2.2%), choroidal detachment due to persistent hypotony (8.9%), hyphema (6.7%) tube exposure (6.7%), cystoid macular edema (4.4%)
Berteloot et al. 2024	23	23.7 (SD 6.9)	13.1 (SD 2.9)	12	44%^α^	91%^α^	Hyphema (4.3%), choroidal effusion (4.3%), hypotony maculopathy (4.3%), shallow anterior chamber (4.3%), corneal decompensation (12.9%), encapsulated bleb (12.9%)
Karapapak et al. 2024	30	38.8 (SD 9.2)	16.1 (SD 3.3)	12	Not specified	86.6%	Choroidal detachment (10.0%), tube exposure (3.3%)
Olgun et al. 2024**	39 PXG29 POAG	34.5 (SD 7.7) in PXG31.9 (SD 7.4) in POAG	13.7 (SD 2.2) in PXG14.8 (SD 3.6) in POAG	12	53.8% PXG68.9% POAG	97.4% PXG86.2% POAG	Hyphema (13.2%), acute hypotonia (13.2%), pupillary membrane (1.4%)
Elhusseiny et al. 2024	25	32.6 (SD 6.1)	13.2 (SD 3.0)	12	24%	80%	Tube exposure (8%), hypotony (4%)
Weber et al. 2024	56	25.4 (range 7–48)	11.3 (range 3–24)	24	52%	89%	Transient hypotony (1.8%), choroidal detachment due to persistent hypotony (7.1%), hyphema (5.4%), cystoid macular edema (3.6%), tube exposure (16.1%), need for PGI explantation due to exposure (8.1%), corneal decompensation (5.4%)
Richardson et al. 2024	50	30.6 (SD 9.8)	12.2 (SD 4.4)	36	48%	92%	Hypotony (8%), corneal decompensation (8%), cystoid macular edema (6%), cataract (12%), hyphema (2%), diplopia (4%), microbial keratitis (4%)
Tan et al. 2024	48	20.6 (SD 6.1)	14.9 (SD 4.1)	36	75% (out of 41 eyes)^α^	85.4% (out of 41 eyes)^α^	Transient hypotony (35.4%), shallow anterior chamber (8.3%), hyphema (10.4%), tube occlusion (8.3%), tube exposure (8.3%), cystoid macular edema (2.1%), corneal decompensation (2.1%)
Khodeiry et al. 2025	42	27.8 (SD 6.4)	15.2 (SD 3.6)	12	42.9%	90.5%	Hypotony (7.1%), hyphema (9.5%), tube exposure (4.8%), corneal decompensation (2.4%)
Weber et al. 2025	34	29.2 (SD 8.1)	14.3 (SD 3.8)	12	50.0%	88.0%	Tube exposure (11.8%), hypotony (8.8%), cystoid macular edema (5.9%)
Studsgaard et al. 2025	76	25.9 (SD 7.3)	13.8 (SD 3.2)	12	46.1%	92.1%	Transient hypotony (10.5%), hyphema (7.9%), tube exposure (5.3%)
Olgun et al. 2025	48 MMC 44 no MMC	33.7 (SD 6.8) MMC 34.2 (SD 7.1) no MMC	15.2 (SD 3.1) MMC 15.8 (SD 3.3) no MMC	12	45.8% MMC 40.9% no MMC	89.6% MMC 86.4% no MMC	Hypotony (6.3% MMC vs 6.8% no MMC), hyphema (8.3% MMC vs 9.1% no MMC), additional surgery requirement (10.4% MMC vs 22.7% no MMC)
Oliver-Gutiérrez et al. 2025	25 PGI 25 BGI	26.8 (SD 7.2) PGI 27.1 (SD 6.9) BGI	13.6 (SD 3.4) PGI 11.9 (SD 4.2) BGI	12	52.0% PGI 48.0% BGI	88.0% PGI 86.0% BGI	Early hypotony (8.0% PGI vs 16.0% BGI), tube exposure (4.0% PGI vs 8.0% BGI)

*POAG* primary open angle glaucoma, *PXG* pseudoexfoliative glaucoma, *MMC* mitomycin C

^α^Success defined as IOP ≤ 18 mmHg

*SD values are provided when available in the source

**For Weber et al. (2023) and Olgun et al. (2024), the table only includes data for IOP ≤ 21 mmHg, in order to be comparable to other studies

In the first report regarding PGI use, Koh et al. analyzed the results of a heterogeneous cohort of 74 eyes undergoing PGI. They observed a mean IOP decrease from 23.1 ± 8.2 mmHg preoperatively to 13.2 ± 3.3 mmHg at 12 months, corresponding to a 42.9% reduction, and reported a 93.2% qualified success rate (IOP ≤ 21 mmHg) at 12 months, with 68.9% achieving complete success (unmedicated IOP ≤ 21 mmHg) [8]. Similarly, Vallabh et al. in 99 eyes, reported a significant reduction in mean IOP from a preoperative value of 28.1 ± 9.0 mmHg to 13.3 ± 4.4 mmHg at 12 months, achieving a 90.1% qualified success rate (IOP < 21 mmHg and > 5 mmHg) at 12 months, with 38.4% attaining complete success (unmedicated IOP < 21 mmHg) [20].

José et al. achieved a mean IOP of 12.5 mmHg at 12 months, a significant reduction from the preoperative mean of 31.4 mmHg, with a qualified success rate of 96.2% and a complete success rate of 58.3% at 12 months [9]. In the first 1-year study of their cohort, Weber et al. reported a decrease in mean IOP from 26.1 mmHg preoperatively to 12.0 mmHg at the end of the follow-up, with qualified and complete success rates (IOP ≤ 21 mmHg) of 95.6% and 73.3% at 12 months, respectively [18]. At 24 months, these rates were 89.3% and 51.8% for qualified and complete success, respectively, using the same IOP criteria [19].

In a comparison between 39 PXG and 29 POAG eyes, Olgun et al. demonstrated satisfactory qualified success (97.4% for PXG and 86.2% for POAG), with a significant reduction in medication burden, after 1 year of follow-up. The reduction in average IOP was similar in the two subgroups, starting from 34.5 mmHg and 31.9 mmHg, and reaching 13.7 mmHg and 14.8 mmHg in PXG and POAG, respectively.

Successively, Tan et al. demonstrated sustained IOP reduction over 2 years, with mean IOP decreasing from 19.8 ± 6.3 mmHg preoperatively to 13.9 ± 3.7 mmHg at 24 months, and found that 71.1% of eyes achieved complete success (unmedicated IOP ≤ 18 mmHg or ≥ 6 mmHg) and further 11.1% achieved qualified success [15]. After the third year of follow-up on 41 eyes, the percentage of eyes reaching complete success reached 75% [16].

Recently, Studsgaard et al. [23] reported outcomes after transitioning from AGV and BGI to PGI at their tertiary center, noting similar efficacy in IOP control with potentially improved safety profiles. Their 12-month data showed a 92% success rate in their cohort of complex glaucoma cases, supporting the clinical utility of PGI across various glaucoma subtypes.

## Results in specific settings

Even if several previous reports included variable percentages of eyes with uveitic glaucoma, Richardson et al. were the first to focus specifically on a cohort of 50 eyes with uveitis, principally anterior and idiopathic. In their study, they reported encouraging results, with a 92% qualified success rate and only one case of prolonged hypotony (2%) at a mean follow-up of 3 years. Overall, mean pre-operative IOP was 30.6 ± 9.8 mmHg using 3.9 ± 0.9 medications, and reduced to 12.2 ± 4.4 under 1.1 ± 1.3 medications [14].

Due to its smaller size, PGI tube was also employed in several studies focusing on pediatric glaucoma, in which ensuring long-term corneal clarity is a fundamental topic. Initially, Elhusseiny et al. reported successful IOP control in three children with refractory childhood glaucoma using PGI, with no postoperative complications observed during nine months of follow-up [11]. In a cohort of 25 patients ranging from 8 months to 16 years affected by congenital, juvenile or secondary glaucoma, Vallabh et al. reported a 48% rate of complete success at 12 months and an 84% of qualified success either at 12- and 24-months follow-up, with significant reduction in terms of medications burden and no changes in visual acuity [17]. Recently, Mendoza-Moreira et al. reported a qualified success rate of 90% at a mean follow-up of 7.7 months in a study involving 10 eyes of 9 children with GFCS. The mean IOP reduction was −14.8 ± 8.73 mmHg, and the number of antiglaucoma medications was significantly reduced from a median of 3.50 to 2.0. Moreover, visual acuity showed a significant improvement during the study period. Only one eye experienced transient numerical hypotony (4 mmHg) without any associated complications [24].

Overall, even if these studies provide promising initial evidence for the use of PGI in managing refractory pediatric glaucoma, longer-term follow-up studies are needed to confirm its efficacy and safety profile in this population.

Another emerging application is in secondary glaucoma following vitreoretinal surgery, as reported by Weber et al. [25] in their recent publication. Their findings suggest PGI is effective in this challenging subset of patients with a qualified success rate of 88% at 12 months. Khodeiry et al. [26] specifically evaluated PGI outcomes in refractory secondary glaucoma, reporting a 90.5% qualified success rate at 12 months. Finally, a novel application reported by Panidou-Marschelke et al. [27] involved successful management with PGI after complicated bleb needling with uveal prolapse into the bleb ten years after trabeculectomy. This case report highlights the utility of PGI in managing challenging post-surgical complications of prior glaucoma procedures.

## Failure rates

Failure rates for the PGI vary across studies, with differing criteria used to define failure and a different range of identified causes. Vallabh et al. reported a 9.3% failure rate at 1 year, defining failure as IOP > 21 mmHg or a < 20% reduction of IOP for two consecutive visits after 3 months. [20] Koh et al. observed a 5.4% failure rate at 1 year, but criteria for failure included also persistent hypotony (IOP < 6 mmHg), need for additional surgery, loss of light perception, or removal of the implant (which was caused by presumed endophthalmitis and recurrent conjunctival erosions). [8] Finally, at 2 and 3 years, Tan et al. documented 17.8% and 14.6% failure rates, respectively, defining failure as IOP > 18 mm Hg or < 6 mm Hg at two consecutive visits after 3 months, reoperation, explantation (due to vitreous-induced tube occlusion in their cohort), or loss of light perception [15, 16].

## Post-operative complications

Complications after PGI surgery can generally be divided into those that occur early, typically within the first three months, and late complications.

Early complications are more frequent and include hypotony, shallow anterior chamber, choroidal effusion, hyphema, tube occlusion, and tube exposure. Hypotony is one of the most commonly observed early complications, with reported rates ranging from 2% to 35.4% in various reports [16, 20]. This variability likely stems from differences in surgical techniques, patient characteristics, and the criteria used to define hypotony. However, many cases of hypotony are transient and resolve spontaneously without requiring intervention [9, 19, 20], while hypotony-induced choroidal effusion occurs less frequently, with rates varying from 0% to 13.3% [10, 12, 19].

A shallow anterior chamber is another frequent finding in the early postoperative period, with reported rates reaching 14.9–22.2% of cases [8, 20]. Most cases are self-limiting and tend to resolve within a few weeks, in line with the occurrence of transient hyphema, which showed reported rates of 4–13.2% [13, 20].

Tube occlusion, or blockage of the tube, can occur due to various factors, such as iris, fibrin, blood, or viscoelastic, and reported rates reach 8.9% [15]. On the other side, early tube exposure has been observed in 4.1% to 6.7% of cases and may require surgical revision [19].

Late complications, emerging after the initial three months, are less common but tend to be more serious. Persistent hypotony is rare, generally around 1–2% of cases, while delayed tube exposure has shown variability throughout the studies. In their research employing fascia lata rather than pericardium for covering PGI, Weber et al. reported tube exposure in 9 eyes (16.1%), 5 of which required PGI explantation and eventual re-implantation in another quadrant [19]. Late corneal decompensation, or loss of corneal clarity, has been observed in 2–12% of cases and is a particular concern in patients with pre-existing corneal endothelial compromise [10, 16]. Finally, cataract formation after PGI implantation has also been reported, though the exact incidence varies across studies.

Less frequent late complications include diplopia and strabismus, cystoid macular edema and sporadic reports of vitreous hemorrhage or retinal detachment.

## Bleb morphology

Recent studies have analyzed the morphology of the bleb of PGI, revealing a specific double-layered aspect. This unique bleb configuration is visible on both ultrasound (US) and magnetic resonance imaging (MRI). Weber et al. noted that 78.6% of eyes in their study exhibited double-layered blebs following PGI implantation, and this characteristic was significantly correlated with lower IOP values. They theorized that the double-layered bleb formation, visible in US, arises from fluid draining both directly through the plate and along its margins, facilitated by the flexibility of the PGI plate. [28] In a MRI analysis, Correia Barão et al. also observed a high prevalence of double-layered blebs (94%) across different GDD types, but notably, PGI devices formed significantly larger blebs overall, particularly extending towards under recti muscles. They hypothesized that the smaller lumen of the PGI might contribute to higher early outflow, leading to more aggressive bleb expansion. The larger blebs observed with PGIs, which elevate the endplate against the conjunctiva, were however hypothesized to potentially increase the risk of erosion or extrusion in the long term [29].

Masdipa et al. [30] recently conducted an in vitro analysis of pressure resistance in the PGI compared to Ahmed ClearPath 250 with and without polypropylene thread inside the tube. Their findings confirmed the inherent flow-restricting properties of PGI’s smaller lumen and demonstrated that inserting polypropylene threads into the tube lumen can effectively modulate pressure resistance, offering a potential technique for customizing flow control.

## Comparison with other Glaucoma drainage devices

The comparative evidence between PGI and other GDDs has expanded significantly with recent publications. Of the 18 studies included in this review, 3 were comparative studies specifically designed to evaluate PGI against established alternatives.

Direct comparative studies between PGI and other commonly used GDDs, such as AGV and BGI, are limited. Nevertheless, indirect comparisons have shown significant reductions in the number of IOP-lowering medications following PGI implantation, comparable to reductions observed with AGV and BGI. Weber et al. reported a greater reduction in the number of employed medications with PGI, compared to the AVB study results for both AGV and BGI [19].

In a recent direct comparison between PGI and BGI, Berteloot et al. found similar success rates of 91% and 89%, respectively, with both devices effectively reducing IOP and the need for medications. Notably, the PGI demonstrated superior IOP control in the early postoperative period (up to one month) compared to the BGI, probably due to the smaller diameter of the PGI tube (127 µm), which acts as a built-in flow restrictor and may help to prevent early hypotony. However, the BGI group eventually achieved lower IOP measurements at 12 months (10.4 ± 4.9 vs. 13.1 ± 2.9 mm Hg) [10]. In terms of safety profile, the PGI was associated with lower numbers of either early and late post-operative complications, even if this difference was not significant in this research [10].

Oliver-Gutiérrez et al. [31] compared the PGI with Baerveldt 350 in a single-center study, reporting comparable IOP reduction and success rates at 12 months. Their analysis found no significant differences in complication rates, though the study was limited by sample size. Failure rates were generally similar between the two (12% failure rate for PGI versus 14% for BGI at 12 months).

Finally, the initial findings of the Paul Ahmed Comparison (PAC) study, a randomized controlled trial comparing the PGI to the AGV in children with refractory glaucoma, showed no statistically significant difference in IOP reduction or medication use between the two devices after one year. This study is ongoing and aims for a three-year follow-up. Mean preoperative IOP were 32.6 ± 6.1 mmHg in the PGI group and 29.8 ± 6.1 mmHg in the AGV group, reducing to 14.9 ± 4.1 mmHg in the PGI group and 15.5 ± 3.5 mmHg in the AGV group at 1 year. The PGI group had a higher mean IOP at 3 months postoperatively, likely due to ripcord stent use, but this difference disappeared over time. Overall success rates, defined as IOP ≤ 21 mmHg, were 92% for the PGI and 84% for the AGV, while qualified success with an IOP ≤ 18 mmHg was 80% for the PGI and 73% for the AGV. In addition, the AGV group had more complications, with two cases of hypotony and one of endophthalmitis, compared to none in the PGI group [21]. Similarly, Bamefleh et al. [32] compared PGI with AGV in managing aphakic glaucoma, finding comparable efficacy with potentially reduced complications in the PGI group, though their report was limited by small sample size.

## Quality assessment and risk of bias

Quality assessment of the included studies using the NIH Quality Assessment Tool for Before-After Studies revealed varying methodological quality (Table 3). Of the 18 studies, 5 (27.8%) were rated as “good,” 10 (55.6%) as “fair,” and 3 (16.7%) as “poor.” Common methodological limitations included:

1. Lack of sample size justification
2. High loss to follow-up in some studies
3. Absence of blinded outcome assessment
4. Heterogeneity in outcome definitions
5. Limited adjustment for potential confounding factors

**Table 3:** Quality assessment of included studies using the NIH Quality Assessment Tool for Before-After (Pre-Post) Studies with No Control Group

Study (First Author, Year)	Clear Research Question	Eligibility Criteria Specified	Representative Sample	All Eligible Participants Enrolled	Sample Size Justification	Intervention Clearly Described	Outcome Measures Clearly Defined	Blinded Outcome Assessment	Loss to Follow-up < 20%	Statistical Methods Appropriate	Multiple Outcome Measurements	Group-Level Interventions	Individual-Level Data Analysis	Overall Quality Rating
Koh et al. 2020	Yes	Yes	Yes	CD	No	Yes	Yes	No	Yes	Yes	Yes	No	Yes	Good
Vallabh et al. 2021	Yes	Yes	Yes	Yes	No	Yes	Yes	No	No	Yes	Yes	No	Yes	Fair
José et al. 2022	Yes	Yes	Yes	CD	No	Yes	Yes	No	Yes	Yes	Yes	No	Yes	Fair
Tan et al. 2022	Yes	Yes	Yes	Yes	No	Yes	Yes	No	Yes	Yes	Yes	No	Yes	Good
Weber et al. 2023	Yes	Yes	Yes	Yes	No	Yes	Yes	No	Yes	Yes	Yes	No	Yes	Good
Berteloot et al. 2024	Yes	Yes	Yes	Yes	No	Yes	Yes	No	Yes	Yes	Yes	Yes	Yes	Good
Karapapak et al. 2024	Yes	Yes	Yes	CD	No	Yes	No	No	Yes	Yes	No	No	Yes	Fair
Olgun et al. 2024	Yes	Yes	Yes	CD	No	Yes	Yes	No	Yes	Yes	Yes	No	Yes	Fair
Elhusseiny et al. 2024	Yes	Yes	CD	CD	No	Yes	Yes	No	No	Yes	Yes	No	No	Poor
Weber et al. 2024	Yes	Yes	Yes	Yes	No	Yes	Yes	No	No	Yes	Yes	No	Yes	Fair
Richardson et al. 2024	Yes	Yes	Yes	Yes	No	Yes	Yes	No	Yes	Yes	Yes	No	Yes	Good
Tan et al. 2024	Yes	Yes	Yes	Yes	No	Yes	Yes	No	No	Yes	Yes	No	Yes	Fair
Khodeiry et al. 2025	Yes	Yes	Yes	CD	No	Yes	Yes	No	Yes	Yes	Yes	No	Yes	Fair
Weber et al. 2025	Yes	Yes	Yes	CD	No	Yes	Yes	No	Yes	Yes	Yes	No	Yes	Fair
Studsgaard et al. 2025	Yes	Yes	Yes	Yes	No	Yes	Yes	No	Yes	Yes	Yes	No	Yes	Fair
Olgun et al. 2025	Yes	Yes	Yes	Yes	No	Yes	Yes	No	Yes	Yes	Yes	Yes	Yes	Fair
Oliver-Gutiérrez et al. 2025	Yes	Yes	Yes	CD	No	Yes	Yes	No	Yes	Yes	Yes	Yes	Yes	Fair
Elhusseiny et al. 2024 (PAC)	Yes	Yes	Yes	Yes	Yes	Yes	Yes	Yes	Yes	Yes	Yes	No	Yes	Good

*CD* Cannot Determine, *PAC* Paul Ahmed Comparison study

Quality Rating Scale:—Good: Study meets most quality criteria, providing reliable evidence with limited potential for bias—Fair: Study meets some but not all quality criteria, with moderate potential for bias—Poor: Study has significant methodological limitations, with high potential for bias

Among the three comparative studies, risk of bias assessment using ROBINS-I identified moderate risk of bias due to potential confounding and selection bias in the non-randomized comparisons. Only the PAC study, being randomized, demonstrated lower risk of bias across most domains.

## Discussion

The Paul Glaucoma Implant has emerged as a promising addition to the armamentarium of glaucoma surgeons, offering a novel approach to managing moderate to advanced glaucoma. Its unique design, featuring a smaller internal tube diameter, aims to reduce the risk of postoperative hypotony while maintaining effective IOP control. Numerous studies have demonstrated the PGI's efficacy in lowering IOP, reducing medication dependence, and achieving both complete and qualified success rates comparable to other established GDDs like the AGV and BGI. The device has also shown promising results in specific settings, such as pediatric glaucoma, offering a potential solution for managing IOP in these challenging cases.

This systematic review of 18 studies including 946 eyes provides the most comprehensive synthesis of PGI outcomes to date. The collective evidence suggests that PGI offers effective IOP control across various glaucoma subtypes with success rates generally comparable to established alternatives. The weighted mean IOP reduction across all studies was 15.2 mmHg, representing an average decrease of 54.7% from baseline values. Complete success rates varied considerably (38.4–75%), likely reflecting differences in definitions and patient populations, while qualified success rates were more consistent and generally exceeded 85%. Even if post-operative adverse events, including hypotony, tube exposure, and corneal endothelial damage, are shared with similar devices, the incidence of hypotony appears to be lower with PGI compared to other non-valved GDD due to its intrinsic structure.

When considering the relative importance of safety versus efficacy in glaucoma drainage devices, the permanent nature of implantable devices and the potential for sight-threatening complications necessitates a safety-first approach. This is particularly relevant for PGI, as its primary design innovation—the smaller inner tube diameter—represents a technical solution specifically addressing the safety concern of postoperative hypotony. The promising early safety profile of PGI, particularly regarding reduced hypotony rates compared to other non-valved implants, represents a potential advantage that warrants further investigation in long-term studies.

A distinctive feature of PGI appears to be its propensity to form double-layered blebs that are significantly larger than those of other GDDs. This morphological characteristic correlates with lower IOP values and may contribute to the device’s efficacy. However, the larger bleb size raises potential concerns about long-term risk of exposure and erosion that will require vigilant monitoring in future studies [29].

The quality assessment revealed substantial methodological limitations in the current evidence base. Most studies were uncontrolled, retrospective case series with moderate risk of bias. Sample sizes were often small, and follow-up periods relatively short, with only three studies reporting outcomes beyond 24 months. Definitions of success and failure varied considerably, complicating direct comparison across studies. These limitations underscore the preliminary nature of the current evidence and highlight the need for more rigorous, long-term comparative studies. Further research, particularly long-term follow-up studies and randomized controlled trials comparing PGI to other GDDs, is warranted to better delineate its optimal role in glaucoma management. As the evidence base continues to grow, the PGI holds the potential to become a valuable tool for ophthalmologists in their pursuit of preserving vision and improving the quality of life for individuals with glaucoma.

## Conclusion

The Paul Glaucoma Implant is emerging as a safe and effective surgical option for treating refractory glaucoma. Its smaller tube diameter and large surface area endplate contribute to efficient IOP control with a potentially lower risk of hypotony. The current evidence base, comprising 18 studies with 946 eyes, demonstrates consistent IOP reduction and high qualified success rates across various glaucoma subtypes and patient populations.

While early comparative data suggest potentially improved safety profiles compared to other GDDs, particularly regarding postoperative hypotony, these findings require validation in larger, long-term randomized controlled trials. The unique bleb morphology of PGI, characterized by double-layered and larger blebs, appears to contribute to its efficacy but raises questions about long-term complications that warrant further investigation.

Based on current evidence, PGI may be considered as an alternative to other GDDs in cases of refractory glaucoma requiring surgical intervention, particularly when postoperative hypotony is a significant concern. The device appears suitable for various glaucoma subtypes, and in pediatric glaucoma, its smaller tube size may reduce the risk of corneal endothelial damage. Tube occlusion techniques should be considered case-by-case, and careful monitoring for tube exposure is essential, particularly given the larger bleb size typically observed with PGI.

Given these methodological limitations and relatively short follow-up periods, clinicians should maintain cautious optimism about PGI’s role in the surgical management of glaucoma. As the evidence base continues to mature, this promising device may establish itself as a valuable addition to the glaucoma surgeon’s armamentarium, potentially offering an improved safety profile while maintaining comparable efficacy to established alternatives.

## References

[1] Schuster AK, Erb C, Hoffmann EM, Dietlein T, Pfeiffer N (2020) The diagnosis and treatment of glaucoma. Dtsch Arztebl Int 117:225 32343668 10.3238/arztebl.2020.0225 PMC7196841

[2] Weinreb RN, Aung T, Medeiros FA (2014) The pathophysiology and treatment of glaucoma: a review. JAMA 311:1901–1911 24825645 10.1001/jama.2014.3192 PMC4523637

[3] Carlà MM, Gambini G, Savastano A, Giannuzzi F, Boselli F, Rizzo S (2024) State Of The Art, Advantages And Drawbacks Of XEN 63 Gel Stent In Glaucoma Surgery. AJO International 100058

[4] Gambini G, Carlà MM, Giannuzzi F, Caporossi T, De Vico U, Savastano A, Baldascino A, Rizzo C, Kilian R, Caporossi A (2022) PreserFlo® MicroShunt: an overview of this minimally invasive device for open-angle glaucoma. Vision 6:12 35225971 10.3390/vision6010012 PMC8883991

[5] Sharma A, Tijerina JD, Bitrian E (2024) Minimally invasive, maximally impactful: minimally invasive glaucoma surgery and the changing glaucoma landscape. Curr Opin Ophthalmol 35:409–414 39082111 10.1097/ICU.0000000000001077

[6] Gupta S, Jeria S (2022) A review on glaucoma drainage devices and its complications. Cureus 14 10.7759/cureus.29072 PMC9554953 36249639

[7] Giovingo M (2014) Complications of glaucoma drainage device surgery: a reviewSeminars in ophthalmology. Taylor & Francis, pp. 397–402. 10.3109/08820538.2014.959199 25325865

[8] Koh V, Chew P, Triolo G, Lim KS, Barton K, Group PGIS (2020) Treatment Outcomes Using the PAUL Glaucoma Implant to Control Intraocular Pressure in Eyes with Refractory Glaucoma. Ophthalmol Glaucoma 3:350-359 10.1016/j.ogla.2020.05.001 10.1016/j.ogla.2020.05.001 32980037

[9] Jose P, Barao RC, Teixeira FJ, Marques RE, Peschiera R, Barata A, Abegao Pinto L (2022) One-year efficacy and safety of the PAUL glaucoma implant using a standardized surgical protocol. J Glaucoma 31:201–205. 10.1097/IJG.0000000000001969 34930872 10.1097/IJG.0000000000001969

[10] Berteloot S, Correia Barao R, Abegao Pinto L, Vandewalle E, Stalmans I, Lemmens S (2024) Treatment outcomes comparing the paul and baerveldt glaucoma implants after one year of follow-up. J Glaucoma 33:594–600. 10.1097/IJG.0000000000002366 38700482 10.1097/IJG.0000000000002366 PMC11319072

[11] Elhusseiny AM, Khodeiry MM, Lee RK, Shaarawy T, Waqar S, Sayed MS (2023) Early experience with the Paul Glaucoma implant in childhood glaucoma: a case series. Clin Ophthalmol 17:1939–1944. 10.2147/OPTH.S414183 37435394 10.2147/OPTH.S414183 PMC10332410

[12] Karapapak M, Olgun A (2024) Efficacy and safety of the paul glaucoma implant in the treatment of refractory primary congenital glaucoma. Jpn J Ophthalmol 68:571–577. 10.1007/s10384-024-01076-0 38935223 10.1007/s10384-024-01076-0 PMC11420279

[13] Olgun A, Karapapak M (2024) Assessing the efficacy of the PAUL Glaucoma Implant in Pseudoexfoliative Glaucoma. Beyoglu Eye J 9:26–32. 10.14744/bej.2024.96729 38504964 10.14744/bej.2024.96729 PMC10944855

[14] Richardson J, Tacea F, Yu J, Yau K, Fenerty C, Au L (2024) The PAUL Glaucoma Implant in the management of uveitic glaucoma-3-year follow-up. Eye (Lond). 10.1038/s41433-024-03527-x 39623106 10.1038/s41433-024-03527-x PMC11933445

[15] Tan MCJ, Choy HYC, Koh Teck Chang V, Aquino MC, Sng CCA, Lim DKA, Loon SC, Chew Tec Kuan P (2022) Two-year outcomes of the Paul Glaucoma implant for treatment of Glaucoma. J Glaucoma 31:449–455. 10.1097/IJG.0000000000001998 35180153 10.1097/IJG.0000000000001998 PMC9148669

[16] Tan MCJ, Ong CW, Aquino MC, Lun KW, Sng CCA, Lim DKA, Loon SC, Koh VTC, Chew PTK (2024) Three-year outcomes of the Paul Glaucoma implant for treatment of Glaucoma. J Glaucoma 33:478–485. 10.1097/IJG.0000000000002369 38506749 10.1097/IJG.0000000000002369 PMC11210944

[17] Vallabh NA, Mohindra R, Drysdale E, Mason F, Fenerty CH, Yau K (2023) The PAUL(R) glaucoma implant: 1-year results of a novel glaucoma drainage device in a paediatric cohort. Graefes Arch Clin Exp Ophthalmol 261:2351–2358. 10.1007/s00417-023-06000-9 36943459 10.1007/s00417-023-06000-9 PMC10028749

[18] Weber C, Hundertmark S, Liegl R, Jauch AS, Stasik I, Holz FG, Mercieca K (2023) Clinical outcomes of the PAUL(R) glaucoma implant: One-year results. Clin Exp Ophthalmol 51:566–576. 10.1111/ceo.14235 37160354 10.1111/ceo.14235

[19] Weber C, Hundertmark S, Stasik I, Holz FG, Mercieca K (2024) Two-year clinical outcomes of the PAUL Glaucoma implant in White patients with refractory Glaucoma. J Glaucoma 33:808–814. 10.1097/IJG.0000000000002457 38940658 10.1097/IJG.0000000000002457

[20] Vallabh NA, Mason F, Yu JTS, Yau K, Fenerty CH, Mercieca K, Spencer AF, Au L (2022) Surgical technique, perioperative management and early outcome data of the PAUL(R) glaucoma drainage device. Eye (Lond) 36:1905–1910. 10.1038/s41433-021-01737-1 34545206 10.1038/s41433-021-01737-1 PMC8450714

[21] Elhusseiny AM, Khaled OM, Chauhan MZ, Sayed MS, Shaarawy T (2024) Initial results of the Paul Ahmed Comparison (PAC) study in refractory childhood glaucoma. Am J Ophthalmol 271:71–78. 10.1016/j.ajo.2024.10.024 39510370 10.1016/j.ajo.2024.10.024

[22] Olgun A, Karapapak M (2025) Impact of mitomycin C on surgical outcomes of PAUL glaucoma implant in neovascular glaucoma: 12-month follow-up results. Eur J Ophthalmol 11206721251313835. 10.1177/11206721251313835 10.1177/11206721251313835 39819179

[23] Studsgaard A, Nielsen SE, Telinius N (2025) One tube for all: 1-year outcomes after transition to Paul glaucoma implant at a tertiary centre. Acta Ophthalmol. 10.1111/aos.17443 10.1111/aos.17443 PMC12069969 39853904

[24] Mendoza-Moreira AL, Voigt AM, Stingl JV, Rezapour J, Wagner FM, Schuster AK, Hoffmann EM (2024) Paul Glaucoma Implant following congenital cataract surgery in a pediatric cohort. J Clin Med 13. 10.3390/jcm13102914 10.3390/jcm13102914 PMC11122222 38792454

[25] Weber C, Schipper P, Walz W, Raming K, Künzel S, Holz FG, Liegl R, Mercieca K (2025) Clinical outcomes of the PAUL glaucoma implant for secondary glaucoma after vitreoretinal surgery. Ophthalmologica 248(2):89–100. 10.1159/000543748 39864421 10.1159/000543748

[26] Khodeiry MM, Hassan AK, Elhusseiny AM, Lee RK, Sayed MS (2025) Outcomes of the Paul Glaucoma implant in refractory secondary Glaucoma. Clin Ophthalmol 19:167–174. 10.2147/OPTH.S505220 39844845 10.2147/OPTH.S505220 PMC11752930

[27] Panidou-Marschelke E, Sokolenko E, Framme C, Binter M (2025) Successful management with Paul Glaucoma Drainage Implant after complicated bleb needling with uveal prolapse into the bleb ten years after trabeculectomy. BMC Ophthalmol 25(1):88. 10.1186/s12886-025-03906-2 39994581 10.1186/s12886-025-03906-2 PMC11853764

[28] Weber C, Weinhold L, Walz W, Petrak M, Holz FG, Liegl R, Mercieca K (2024) Sonographic bleb visualisation after PAUL glaucoma implant surgery. Br J Ophthalmol. 10.1136/bjo-2024-326168 39689979 10.1136/bjo-2024-326168

[29] Correia Barao R, Berhanu D, Bernardo Matos D, Barata AD, Sousa R, Abegao Pinto L (2023) Bleb morphology of glaucoma drainage devices on magnetic resonance imaging. Acta Ophthalmol 101:789–796. 10.1111/aos.15668 37066864 10.1111/aos.15668

[30] Masdipa A, Kaidzu S, Tanito M (2025) In Vitro analysis of pressure resistance in the Paul Glaucoma Implant and Ahmed ClearPath 250 with and without polypropylene thread inside the tube. Transl Vis Sci Technol 14(1):2. 10.1167/tvst.14.1.2 39774608 10.1167/tvst.14.1.2 PMC11717134

[31] Oliver-Gutiérrez D, Segura-Duch G, Ávila-Marrón E, Arciniegas-Perasso CA, Duch-Tuesta S (2025) Paul versus Baerveldt 350 glaucoma drainage implants: one-year comparative analysis. Indian J Ophthalmol 73(Suppl 2). 10.4103/IJO.IJO_2595_24 10.4103/IJO.IJO_2595_24 PMC12013288 39982092

[32] Bamefleh D, Alabduljabbar K, Alkhodier A (2025) Comparative outcomes of Ahmed glaucoma valve implant and Paul glaucoma implant in managing aphakic glaucoma: a case report. Int J Surg Case Rep 130:111316. 10.1016/j.ijscr.2025.111316 40267612 10.1016/j.ijscr.2025.111316 PMC12047580

